# The Gap Between Clinical Research and Standard of Care: A Review of Frailty Assessment Scales in Perioperative Surgical Settings

**DOI:** 10.3389/fpubh.2016.00150

**Published:** 2016-07-21

**Authors:** Nicoleta Stoicea, Ramya Baddigam, Jennifer Wajahn, Angela C. Sipes, Carlos E. Arias-Morales, Nicholas Gastaldo, Sergio D. Bergese

**Affiliations:** ^1^Department of Anesthesiology, The Ohio State University Wexner Medical Center, Columbus, OH, USA; ^2^Medical School, The Ohio State University College of Medicine, Columbus, OH, USA; ^3^Medical School, Ohio University Heritage College of Osteopathic Medicine, Dublin, OH, USA; ^4^Department of Neurological Surgery, The Ohio State University Wexner Medical Center, Columbus, OH, USA

**Keywords:** frailty, geriatric population, surgical outcomes, perioperative surgical home, assessment scale

## Abstract

The elderly population in the United States is increasing exponentially in tandem with risk for frailty. Frailty is described by a clinically significant state where a patient is at risk for developing complications requiring increased assistance in daily activities. Frailty syndrome studied in geriatric patients is responsible for an increased risk for falls, and increased mortality. In efforts to prepare for and to intervene in perioperative complications and general frailty, a universal scale to measure frailty is necessary. Many methods for determining frailty have been developed, yet there remains a need to define clinical frailty and, therefore, the most effective way to measure it. This article reviews six popular scales for measuring frailty and evaluates their clinical effectiveness demonstrated in previous studies. By identifying the most time-efficient, criteria comprehensive, and clinically effective scale, a universal scale can be implemented into standard of care and reduce complications from frailty in both non-surgical and surgical settings, especially applied to the perioperative surgical home model. We suggest further evaluation of the Edmonton Frailty Scale for inclusion in patient care.

## Introduction

Frailty in elderly populations can be defined as a “clinical state in which there is an increase in an individual’s vulnerability for developing increased dependency and/or mortality when exposed to a stressor (1).” Rose et al. concluded that the score of Edmonton Frail Scale (EFS) increased toward a higher frail state, an average of 0.22 points for every year the sample population increased in age (2). Frailty in patients is becoming even more apparent as the United States’ “baby boomer” generation ages. It is projected that, by 2030 in the United States (U.S.), there will be 61 million people ages 66–84 and 9 million people over the age of 84, placing a large portion of the population in a high frail state (3). The relationship between frailty and increased morbidity and mortality presents a need for an appropriate assessment tool that optimally quantifies frailty in the clinical and perioperative setting (4).

Although the concept of frailty is widely acknowledged, there is still no consensus regarding the criteria to establish a universal frailty assessment tool. Physical frailty has historically been defined using either a frailty index or a combination of five factors: general weakness, decreased endurance, weight loss, decreased activity, and unstable gait leading to falls (2, 5). Of late, however, frailty has been emerging as a multidimensional concept, including cognitive function and psychosocial factors (6). The FRAIL scale, Cardiovascular Health Study Frailty Screening Measure (CHS), Clinical Frailty Scale, Groningen Frailty Indicator (GFI), Tilburg Frail Indicator (TFI), and EFS are a few of the frailty assessments that have been frequently mentioned in the literature as valid measures of frailty (Table 1) (7). However, there is a gap between the validity of those scales obtained through research and their implementation into clinical practice. Therefore, this review seeks to assess the gap and the feasibility to include comprehensive frailty assessment in standard of care practices.

**Table 1:** **Chart of scales reviewed with corresponding criteria evaluated and scoring scale**.

Scale	Criteria measured	Score
FRAIL scale	Fatigue, resistance, ambulation, illnesses, and loss of weight	0–5
Cardiovascular Health Study Frailty Screening Measure	Shrinking (weight loss), weakness, poor endurance and energy, slowness, and low physical activity level	0–4
Clinical Frailty Scale	Clinical judgment-based assessment of frailty (robust health → total reliance on others)	0–7
Groningen Frail Indicator	Four domains: physical, cognitive, social, and psychological	0–15
Tilburg Frail indicator	Physical health, unexplained weight loss, difficulties in walking, balance, strength in hands, physical tiredness, eyesight, and hearing impairments, cognition, depressive symptoms, anxiety and coping, living alone, social relationships, and social support	0–15
Edmonton Frail Scale	Cognition and balance and gait, mood, functional independence, medication use, social support, nutrition, health attitudes, continence, burden of medical illness, and quality of life	0–17

Currently, there is an increased tendency to use frailty assessments in the surgical setting in order to predict perioperative outcomes in geriatric populations. The perioperative risk is assessed by the American Society of Anesthesiologists (ASA) Classification of Physical Status based on patient systemic diseases with no reference to individual frailty state (8). Dasgupta et al. demonstrated the clinical value of frailty assessments through higher EFS scores in surgical patients over 70 years old correlated with longer length of hospital stay, less likelihood of being discharged, and more postoperative complications (9).

Frailty syndrome studied in geriatric patients is responsible for an increased risk for falls, and increased mortality (10).

A standard frailty assessment tool combined with the ASA Classification of Physical Status would better determine the overall risk for perioperative complications in geriatric patients.

A review of literature published between 2011 and 2016 was conducted accessing PubMed, Google Scholar, and Cochran Library sources and using the following key words: Frailty, Geriatric Population, Surgical Outcomes, Perioperative Surgical Home (PSH), and Assessment Scale.

Our goal was to assess the validity and reliability of different standardized frailty scales and the feasibility of implementing them in a clinical setting.

### FRAIL Scale

The FRAIL scale computes frailty based on five factors, such as fatigue, resistance, ambulation, illnesses, and loss of weight, and is scored on a scale of 0–5 points with the presence of a factor equaling one point (11). In 2012, Morley et al. showed that the scale has been validated for populations of African American patients aged 49–65 years. The study (2012) concluded that a higher FRAIL scale score correlates with greater difficulty with activities of daily living (ADL), ambulation, and physical activity (11). In another similar study, Lopez et al. demonstrated the validity of the FRAIL scale for a group of 8646 Australian women aged 74–82 years. A FRAIL score of more than 2 out of 5 was associated with an increased risk of morbidity and mortality (4).

The FRAIL scale has been extended to chronic dialysis patients in rural communities being preferred to the Strawbridge questionnaire, EFS, Groningen Frail Indicator, G8 questionnaire, and TFI (12).

### Cardiovascular Health Study Frailty Screening Measure

The CHS is based on several factors determined to encompass the frailty phenotype including: shrinking (weight loss), weakness, poor endurance and energy, slowness, and low physical activity level (13). Participants meeting criteria for three or more factors are deemed clinically frail. Those with one to two factors are classified as the intermediate frailty group, while those with no (0) factors are classified as not frail (13). Fried et al. define shrinking by unintentional weight loss of ≥10 pounds within last year or >5% body weight at follow-up. Weakness is described by grip strength within the lowest 20% of the population after statistical standardization for gender and body mass index (13). Poor endurance and energy is determined by self-reported exhaustion collected by using the Center for Epidemiological Studies Depression (CES-D) scale (14). Slowness is assessed by the time required to walk 15 feet, and is defined by those with walking times in the top 20% of the population after statistical standardization for gender and standing height. Low physical activity is determined by those within the lowest quintile of physical activity for each gender (13).

The CHS frailty criteria method has been shown to be a valid measure of frailty in various populations. Fried et al. (13) considered a study population of men and women ≥65 years of age with a variety of prevalent diseases at baseline. Of those participants, 58% were female and 15% were African American. The study found that subjects determined to be frail at baseline by the CHS frailty criteria were shown to have a sixfold (18%) increase in mortality at 3 years compared to those classified as non-frail (3%) (13). At 7 years, patients deemed frail at baseline correlated with a threefold higher mortality (43%) compared to the non-frail group (12%) (13). Those classified as frail by the CHS frailty criteria were also shown to be at increased risk of “incident falls, worsening mobility or ADL disability, hospitalization, and death.” (13) Furthermore, there was a correlation between frailty phenotype and poor socioeconomic status, African American race, and comorbidities (13).

The analysis of Women’s Health and Aging Studies (WHAS) I and II data set applied for CHS concluded that frail women are at sixfold higher risk of mortality and 10-fold higher risk of disabilities in performing instrumental activities of daily living (IADL) and/or ADL and nursing home entry (15). The CHS is functional for predicting mortality as well as disability and, therefore, is a valid measure of frailty (13, 15).

### Clinical Frailty Scale

The Clinical Frailty Scale is a clinical judgment-based assessment of frailty. The scale consists of seven different measures, with the lowest score (1) indicating those with “robust health” and the highest score (7) applying to patients with “complete functional dependence on others.” (16) The intermediate scores (2–6) cover the following categories: well, but with reduced fitness compared to category 1 (2); well, with adequately treated comorbid disease (3); apparently vulnerable (4); mildly frail (5), and moderately frail (6) (16).

The participants of the Canadian Study of Health and Aging (CSHA) consisted of men and women ≥65 years of age, representing three different populations: 210 (9.1%) participants living in institutional facilities, 1326 (57.5%) individuals still living at home or elsewhere in the community and diagnosed with cognitive impairment, and 769 (33.4%) living within the community with no cognitive impairment (16). The study showed that there was a 21.2% significant increase in mortality (*p* = 0.05) and 23.9% increase in admission into institutional care (*p* = 0.05) with every one-category increase of the Clinical Frailty Scale (16). The CSHA also showed that the Clinical Frailty Scale more accurately predicted the mortality risk at 18 and 70 months than individual measures of comorbidities, cognition, or function (16).

### Groningen Frail Indicator

The Groningen Frail Indicator is a frailty assessment consisting of a 15-item list that covers four functional domains: physical, cognitive, social, and psychological (17). A score from 0 to 15 is given after completion of the GFI, with moderate to severe frailty state indicated by a score ≥4 (17). The GFI has been validated in two formats: professionally administered and self-report version (17).

Peters et al. looked at the feasibility, reliability, and construct validity of the self-assessment version of the GFI (17). Her study population included individuals 65 years or older able to fill out self-administered questionnaires, and excluded severely cognitively impaired and/or extremely ill patients (17). In this study, the GFI was shown to have an internal consistency, or reliability, of 0.68 (17). GFI scores were also of statistical significance (*p* = 0.05) among various groups within the population, categorized by demographic and disease/disorder characteristics, indicating group validity (17). The frail population was characterized by higher levels of case complexity, disability, and lower quality of life and life satisfaction (17).

In a similar study, GFI validity and characteristics associated with frailty were assessed in a population of adults 65 years or older with minimal to no cognitive impairment (18). Among the various demographics within the study population, males and the eldest study participants had higher median GFI scores (18). In addition, the elderly who were single and obese with low socioeconomic status, multiple comorbidities, and greater use of healthcare resources also received significantly higher median GFI scores (18). Frail individuals scored worse on the following assessed measures, including case complexity, cognition, feelings and emotions, psychological problems, quality of life, stress, and well-being. In addition, physical and psychological morbidity was more prevalent in the frail population (18).

### Tilburg Frail Indicator

The TFI is a self-reported frailty questionnaire accounts for the physical, psychological, and social contributors of frailty (19). The physical aspect of the questionnaire asks about “physical health, unexplained weight loss, difficulties in walking, balance, strength in hands, physical tiredness, eyesight, and hearing impairments.” (19) The psychological aspects considered are “cognition, depressive symptoms, anxiety and coping.” (19) Lastly, the social aspects considered in the TFI are “living alone, social relationships, and social support.” (19) The TFI is scored on a 0–15 scale with a score of ≥5 recognized as frail (19).

Higher scores on the TFI scale have been shown to correlate with greater disability in ADLs in individuals with acute coronary syndrome (ACS). Uchmanowicz et al. tested the TFI in a sample of 135 patients with ACS who had had a ST segment elevation myocardial infarction (STEMI) or non-ST segment elevation myocardial infarction (NSTEMI) (20). A TFI score ≥5 indicated increased disability in ADLs in this study population (20). The TFI has also been shown to be predictive of difficulties in ADLs and IADLs in patients over the age of 65 with a 2.5-year follow-up (21). In particular, the physical contributors measured regarding weakness, stamina, speed of movement, and physical activity were able to predict disability in ADLs and IADLs (21).

The TFI was originally created in the Dutch language but has been repurposed into English, Portuguese, Danish, and Polish and shown to be both reliable and valid in these five formats. The German translation was tested in German-speakers aged 64–91 years in sample sizes of 35 and 175 and showed preliminary validity, test–retest reliability after 5 months, and internal consistency in this sample (19).

### Edmonton Frail Scale

The EFS looks at frailty based on 10 components; two of the components have the patient perform activities to determine cognition and balance and gait. The remaining eight components are determined by an interview and examine the patient’s self-reported “mood, functional independence, medication use, social support, nutrition, health attitudes, continence, burden of medical illness and quality of life.” (22) The addition of social support as a determinant of frailty is a feature unique to a few scales including the EFS.

The EFS is administered with a clear structure to evaluate frailty in less than 5 min and is scored from 0 (not frail) to 17 (extremely frail) making the results easy to interpret in a clinical setting (22). Additionally, the validity and reliability is equivalent when the EFS is administered by individuals without formal medical training (22). The use of the EFS has also been extended to specific patient populations, such as elderly patients with ACS (23). In a sample of 183 ACS patients over the age of 65, Graham et al. found that higher EFS scores correlated with greater mortality (23).

The EFS has been shown to be predictive the length of hospital stay in an acute setting (24). However, frailty score did not accurately predict the length of the hospital stay or ambulation in a sample of 75 elderly patients in subacute care (25).

## Comparison of Scales

Some frailty assessment tools have been compared against each other in specific patient populations. The CHS Frailty Screening Measure and FRAIL scale were compared in a sample of 4000 men and women in Hong Kong >65 years old (26). This study excluded individuals who had other comorbidities that could predict a projected mortality in the next 4 years and individuals who could not walk independently. Woo et al. found both scales to be predictive of morbidity and mortality (26). Both the CHS and FRAIL scales were more specific and less sensitive at greater degrees of frailty (26). The specificity was >90% for 4-year mortality in individuals with greater frailty in both the CHS and FRAIL scales [24]. However, the CHS Frailty Screening Measure is less discriminatory in its definition of frailty and categorized more individuals as prefrail or frail than the FRAIL scale, indicating a higher sensitivity in the CHS Frailty Screening Measure (26).

In a 2014 study of 998 African Americans aged 49–65 years in St. Louis, MO, USA, the FRAIL scale was found to be more predictive of difficulty with ADLs and IADLs within 3 years than the CHS (27). Both scales were equivalently predictive of increased difficulty with ADLs and IADLs within 9 years. However, the study also found that a categorization of “frail” on the FRAIL scale predicts mortality within 9 years, whereas mortality within 9 years in patients classified as “frail” with CHS was not accurately predicted (27).

Woo et al. considered the greatest contrast between the FRAIL scale and the CHS to be the ease of implementation into clinical practice (26). The CHS measure may be less ideal for a clinical practice because it requires population reference values and is a more time-consuming test to perform (26). The FRAIL scale is much easier to implement bedside because it only consists of five questions, whereas the CHS requires timed physical assessments (26).

The GFI and TFI were also compared in a 2012 study of 430 participants >70 years old in the Netherlands (28). Over a 1-year follow-up, the GFI was found to be more sensitive (71%) than the TFI (62%) for predicting disability (28). The TFI had higher specificity (65%) than the GFI (55%) for predicting hospital admissions, but both tests have nearly equivalent sensitivity with respect to hospital admissions. However, neither test was shown to be statistically significant for predicting frailty (28). The GFI and TFI have been shown to be equally sensitive for prediction of mortality (28). Overall, Daniels et al. recommended the GFI because of the better predictability of future disability than the TFI (28).

Theou et al. evaluated multiple frailty scales for their ability to assess frailty and capacity to predict all-cause mortality (29). The studied frailty scales included the following, some of which were modified to account for data limitations: GFI, Tilburg Frailty Indicator, Clinical Frailty Scale, CHS, EFS, and the FRAIL scale (29). To assess these scales, a secondary analysis of the Survey of Health, Ageing and Retirement in Europe (SHARE) was performed, resulting in a study population of participants aged 50–104 years and representative of 11 different European countries (29). The unweighted SHARE-EFS was found to be one of the most predictive for all-cause mortality (29). Overall, Theou et al. recommend the use of the SHARE-EFS because it was shown to have the best predictive ability of all-cause mortality (29).

## Discussion

### Recommendations of Frailty Scales in Clinical and Surgical Settings

Despite the evidence that frailty can predict morbidity and mortality, assessment of frailty is hardly utilized in clinical practice today (30). In regard to ease of clinical use, the EFS and the FRAIL scale could be implemented into the hospital and perioperative setting (22). The EFS does not require a comprehensive geriatric assessment prior to administration and can be completed in less than 5 min, making it ideal for a clinical setting. The FRAIL scale consists of only five questions, making it time-efficient to utilize in a clinic with little disruption of the workflow for clinicians.

Considering the application of a multidisciplinary approach to frailty, the EFS and Groningen Frail Indicator are likely candidates for clinical use. Frailty requires a more comprehensive evaluation because it is “multidimensional, heterogeneous, and unstable.” (22) The EFS looks at multiple factors to determine frailty including: “mood, functional independence, medication use, social support, nutrition, health attitudes, continence, burden of medical illness and quality of life.” (22) The GFI considers physical, cognitive, social, and psychological effects on frailty.

Because the FRAIL scale, EFS, and GFI are best suited for the non-surgical setting, it is important for future research to examine their function in the perioperative setting. Despite the benefits of the multidisciplinary approach of the GFI, Bras et al. found that elderly patients, undergoing head and neck surgery and were preoperatively classified as frail by the GFI, were not at increased risk of postoperative complications (31). However, the “health problems” aspect of the GFI questionnaire was predictive of postoperative complications (*p* = 0.020), traditionally can be measured by the ASA score (31). By contrast, a different study (2014) looked at geriatric patients undergoing surgery for gastric adenocarcinoma found that the preoperative GFI frailty status was significantly associated with mortality (32). With conflicting information regarding the value of the preoperative GFI assessments in predicting postoperative complications, further studies should attempt to better assess the overall predictive validity of the GFI in the surgical setting.

Per Jones et al., a history of falling in the 6 months prior to an elective surgery is a predictor of one or more postoperative complications and 30-day readmission rate (33).

A Danish observational study conducted in patients older than 85 years undergoing fast track joint replacement concluded that of 90-day readmission (*n* = 98) the most frequent cause was falling – 24.3% (*n* = 18). The study emphasized the importance of identifying the use of preoperative walking aids and a history of anemia as predictors of readmission (34).

A prospective cohort study published by Nolan et al. examined the impact of Time up and Go test on rehabilitation outcomes of frail older patients undergoing inpatient rehabilitation. CFS, Elderly Mobility Scale, and Falls Efficacy Scale assessments indicated that CFS predicted mobility “markers,” length of stay, and dependency (35).

An increasing body of evidence supports the use of frailty scales to identify frail older individuals living at home. A Dutch home-based cognitive behavioral intervention proved to reduce individual’s concerns about falls and disability. Groningen Activity Restriction Scale (GARS), Falls Efficacy Scale-International Avoidance Behavior (FES-IAB), and ADL were instrumental in identifying study population (36).

Future research should compare different frailty assessment tools because of the limited research in perioperative settings. Further studies should determine whether there is a statistically significant correlation between frailty and adverse surgical outcomes. Given our evaluation of the above frailty scales, we recommend a focus on the FRAIL scale and EFS. Both FRAIL scale and EFS offer a time-efficient but effective measure of frailty in clinical settings that has potential to determine the best predictor of multiple surgical complications and postoperative outcomes.

### Novel use for Frailty Scores within a Perioperative Surgical Home Model

Frailty assessments can further be utilized in newer clinical patient-centered models, such as the PSH to allow for better development of patient-specific management plans. The PSH is an anesthesiologist-led, multidisciplinary team-based care model endorsed by the ASA that provides patients with perioperative continuity of care for 30 days after their surgical intervention (37–39). Including an initial, standardized, and categorical assessment of frailty at the outset of treatment provides a baseline for risks of perioperative complications. The five physical factors evaluated in frailty assessment of general weakness, decreased endurance, weight loss, decreased activity, and unstable gait can help identify patients’ risks for increased dependency over the course of their life. Overlooking the level of physical strength at the outset of patient treatment can attenuate attention to fall risk and other deficiencies in self-care as well as the opportunity to provide interventional physical and occupational therapy at the most effective time. However, increasing awareness by periodically assessing patients’ progress with the EFS can continually adjust treatment plans. Frailty scales can aid the PSH model by more accurately predicting the patients’ postoperative complications in order to implement risk-reducing interventions.

By including initial evaluations of frailty and foreseeing perioperative complications, there will be a decrease in patient risk for complication that will improve clinical outcomes, increase patient-centeredness, and decrease perioperative costs (37, 39–41). Patient-centeredness and patient-specific needs are addressed by the Edmonton Frailty Scale; the EFS encompasses “mood, functional independence, medication use, social support, nutrition, health attitudes, continence, burden of medical illness and quality of life” thereby creating a plan that addresses a patient’s specific needs and instilling a sense of ownership of a recovery plan (22). The EFS also assesses a patient’s balance and gait, which directly impacts likelihood for falls. If the use of the EFS were implemented at the outset of patient treatment to periodically evaluate changes in these characteristics over the course of care, interventions could be applied as soon as declines are identified in order to reduce likelihood of a fall. By addressing directly these deficits in balance and gait as a standard of care in elderly patients and especially those considered frail, fall rates could drastically decline as a result of advanced intervention versus post-incident therapy.

The ESF included in the PSH could enhance patient accountability of plans of care to extend beyond treatment given by a medical professional to patient-centered care and increase adherence to medical recommendations and advice. Standardized Clinical Assessment and Management Plans (SCAMPS) have shown great reductions in costs per patient for total medical expenses, showing up to 50% cost reduction (42).

## Conclusion

Given the fact that the elderly population in the U.S. continues to grow exponentially, it is of utmost importance for frailty assessments to be implemented into clinical practice in order to predict clinical outcomes and to intervene appropriately in patient care. A consensus must be made to eliminate variations in definitions of frailty in order to create a universal tool able to assess the status of patients during surgical consults, admissions, and peri/postoperative care. Considering the recent success of PSHs, we recommend that frailty assessments should be incorporated into the perioperative setting, in order to establish a detectable patient characteristic that is associated with an increased risk of adverse postoperative outcomes and to intervene appropriately.

## Author Contributions

The authors state equal contribution in the elaboration of this manuscript.

## Conflict of Interest Statement

The authors declare that the research was conducted in the absence of any commercial or financial relationships that could be construed as a potential conflict of interest.
